# DFT Calculations of ^31^P NMR Chemical Shifts in Palladium Complexes

**DOI:** 10.3390/molecules27092668

**Published:** 2022-04-21

**Authors:** Svetlana A. Kondrashova, Fedor M. Polyancev, Shamil K. Latypov

**Affiliations:** Arbuzov Institute of Organic and Physical Chemistry, FRC Kazan Scientific Center of RAS, 420088 Kazan, Russia; kondrashovamail@gmail.com (S.A.K.); fedor.polyantsev@yandex.ru (F.M.P.)

**Keywords:** DFT calculations, NMR chemical shifts, palladium complexes, phosphorus

## Abstract

In this study, comparative analysis of calculated (GIAO method, DFT level) and experimental ^31^P NMR shifts for a wide range of model palladium complexes showed that, on the whole, the theory reproduces the experimental data well. The exceptions are the complexes with the P=O phosphorus, for which there is a systematic underestimation of shielding, the value of which depends on the flexibility of the basis sets, especially at the geometry optimization stage. The use of triple-ζ quality basis sets and additional polarization functions at this stage reduces the underestimation of shielding for such phosphorus atoms. To summarize, in practice, for the rapid assessment of ^31^P NMR shifts, with the exception of the P=O type, a simple PBE0/{6-311G(2d,2p); Pd(SDD)}//PBE0/{6-31+G(d); Pd(SDD)} approximation is quite acceptable (*RMSE* = 8.9 ppm). Optimal, from the point of view of “price–quality” ratio, is the PBE0/{6-311G(2d,2p); Pd(SDD)}//PBE0/{6-311+G(2d); Pd(SDD)} (*RMSE* = 8.0 ppm) and the PBE0/{def2-TZVP; Pd(SDD)}//PBE0/{6-311+G(2d); Pd(SDD)} (*RMSE* = 6.9 ppm) approaches. In all cases, a linear scaling procedure is necessary to minimize systematic errors.

## 1. Introduction

Transition metal (TM) complexes are catalysts for a number of reactions that are important from the point of view of practical applications [1,2,3,4]. The rational design of such systems requires information about the structure, type of coordination, and electronic structure as well. To this end, NMR chemical shifts (CS) of atoms directly coordinating the metal are sensitive to the electronic structure and, thus, can be used to obtain hidden information about the electronic state of the complex. Therefore, one needs to have a tool that allows the calculation of NMR shifts based on the principles of quantum chemistry. However, in the case of TM complexes, this is not a trivial task, and until recently, there was no such computational method that could be considered safe and reliable. This is due to the fact that in TM the requirements to account for electron correlation effects [5,6] sharply increase. Moreover, in the case of heavier metals, it may be necessary to take into account relativistic effects as well [7,8,9].

Nevertheless, it has recently been shown [10,11,12] that for nickel complexes, the ^13^C and ^31^P CSs can be calculated within the non-relativistic approximation at the Kohn–Sham (KS) density functional theory [13]. Moreover, the approach allowed several anomalies to be explained with the interpretation of NMR data [10] and to revise some structures [11].

From the point of view of catalysis and catalyst development, palladium- and platinum-based complexes play important roles [14,15,16,17,18,19,20,21,22,23]. Therefore, in order to deal with such systems, it is necessary to have a similar tool to calculate ^31^P CSs in the complexes of these metals. In this case, however, there is a certain pessimism, since it is believed that for *4d* and, especially for *5d* metals, relativistic effects may need to be taken into account [8]. Unfortunately, fully relativistic calculations for such systems are still difficult due to the enormous demands on computational resources. In this regard, it is interesting to see whether it will be sufficient to take these effects into account, at least within the framework of relatively simple scalar approximations using the quasi-relativistic effective core potential (ECP) [24]. Therefore, at this stage, we try to evaluate the scopes and limitations of density functional theory (DFT) methods for estimating ^31^P CSs in palladium complexes using this approach to describe the electronic structure of the metal. In fact, the present study was motivated by practical needs and the main goal is to propose a simple DFT method that could be safely applied to calculate ^31^P NMR shifts in Pd complexes.

## 2. Results and Discussion

### 2.1. General Overview

To calculate the ^31^P NMR shifts in Pd complexes, a combination similar to the one used in nickel complexes was applied as an initial approximation [11]—namely, the PBE0 functional with the Pople’s basis set of double-ζ quality (6-31+G(d)) on elements for geometry optimization and the basis set of triple-ζ quality (6-311G(2d,2p)) for shielding calculation—while ECP SDD was applied to describe Pd (PBE0/{6-311G(2d,2p); Pd(SDD)}//PBE0/{6-31+G(d); Pd(SDD)}; hereafter, this combination will be referred to as comb_1. To assess the quality of the calculation, Pd complexes based on organophosphorus ligands (39 in total, structures with numbering in Figures S1–S4 in the Supporting Information (SI)), which represent almost all known complexes of this type and have ^31^P CSs covering the wide range of possible values (from −50 to 200 ppm), were used as components of a test set. Calculations were carried out within the framework of the GIAO method [25], all ^31^P CSs were referenced to H_3_PO_4_, and the linear scaling procedure was applied [26,27,28] with coefficients similar to that for nickel complexes [11]. To begin with, the calculations were carried out without taking into account the solvent effects (Table S1).

**Figure 1:**
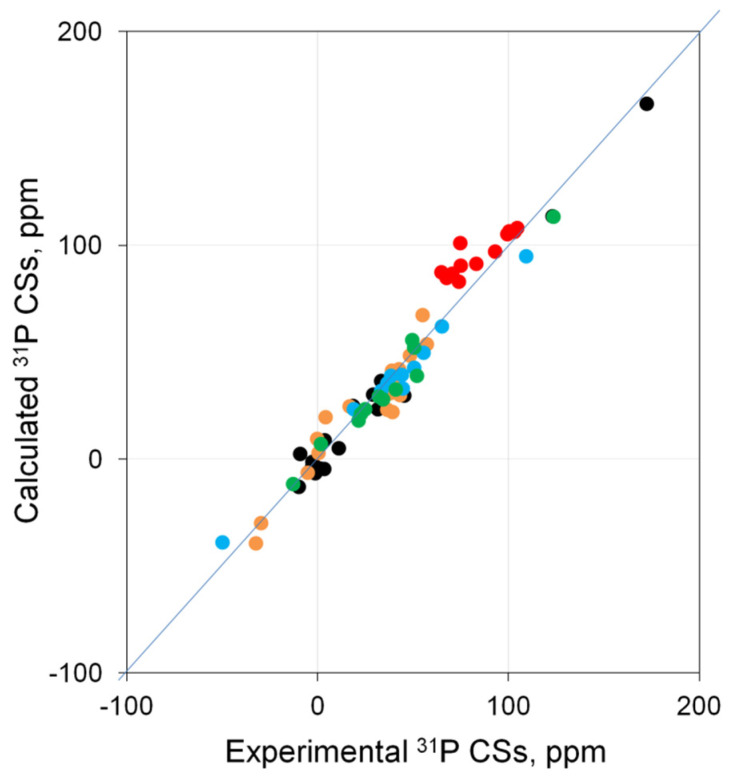
Correlation of calculated (PBE0/{6-311G(2d,2p); Pd(SDD)}//PBE0/{6-31+G(d); Pd(SDD)}) vs. experimental ^31^P CSs for palladium complexes **1**–**39**. Complexes based on σ-donor ligands (●), charged palladium complexes based on σ-donor ligands (●), complexes containing P=O groups (● are the P=O phosphorus, ● are other phosphorus atoms in these types of complexes), and complexes based on π-donor ligands (●).

It was found that, in general, for most of the complexes, the theory reproduces ^31^P CSs well, and there is a correlation between the calculated and experimental data close to linear (Figure 1).

A more detailed analysis allowed us to estimate the quality of calculations for each of the types of complexes under consideration. Specifically, in Pd complexes based on σ-donor ligands (Figure S1), the calculated ^31^P CSs correlate well with the experimental data (● in Figure 1, *R*^2^ = 0.982). In charged palladium complexes based on σ-donor ligands (Figure S2), the situation is somewhat more complicated. Here, the best agreement of the calculation with the experiment is achieved (1) for systems with a weakly coordinating counterion (BF_4_) if the counterion is not taken into account in the calculation; (2) for systems with a strongly coordinating counterion (Cl and Br) to take them into account if they complement the complex to a square–planar; otherwise, as with a weak counterion, it is necessary to carry out the calculation without counterion. With this approach, for such systems, there is good agreement between the calculation and experiment (*R*^2^ = 0.988, ● in Figure 1). The most important finding is that the systematic deviation of the calculated ^31^P CS from the experimental ones is observed in Pd complexes based on ligands containing P=O groups (Figure S3). Specifically, there is an overestimation by ~15–30 ppm for CSs of only P=O phosphorus atoms (● in Figure 1). At the same time, for this type of phosphorus, the dependence is also close to linear. As for palladium complexes based on π-donor ligands (Figure S4), the theory reproduces the experiment well (*R*^2^ = 0.973, ● in Figure 1). Thus, it can be argued that, at the PBE0/{6-311G(2d,2p); Pd(SDD)}//PBE0/{6-31+G(d); Pd(SDD)} level, a good agreement can be reached between the calculated and experimental ^31^P NMR shifts for most complexes. In principle, there are certain problems only with the P=O type of phosphorus.

### 2.2. Optimization of the Computational Approach

In an attempt to resolve this problem, the main factors that can affect the results of the calculation were considered. These are the medium effects, the impact of high-spin states, the influence of the quality of basis set/functional, the possible impact of relativistic effects, etc. Of the possible effects, one that is easily verifiable is the effect of a solvent. However, calculations taking into account the solvent within the polarizable continuum model (PCM) do not lead to improvements for the P=O phosphorus atoms (Table S1). Moreover, contributions due to high-spin states can be also excluded, since these molecular systems are very high in energy (35–60 kcal/mole, Table S3).

**Figure 2:**
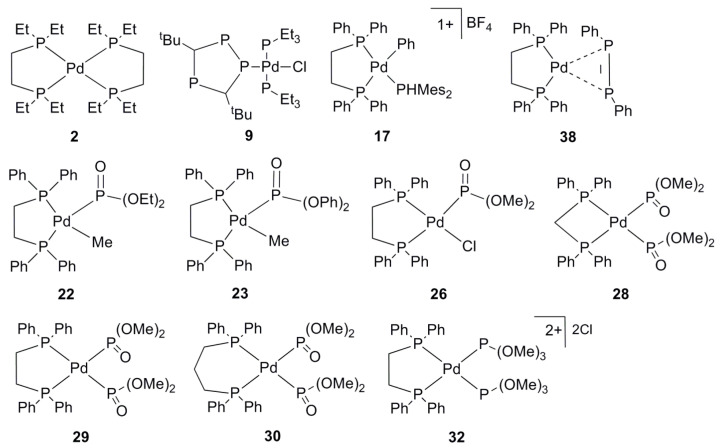
“Training” set of palladium complexes.

Thus, there is no “simple” way to correct the deviation for the P=O type of phosphorus. In order to create a cost-effective method, we fixed the functional to the PBE0 and screened different basis-set/functional combinations so as to obtain adequate agreement between calculated and experimental ^31^P shifts. This requires a detailed systematic analysis, which is more convenient to carry out on a smaller number of “training” models, including both problematic complexes (**22**, **23**, **26**, **28**–**30**, **32**) and several normal systems (**2**, **9**, **17**, **38**) with different types of ligands (σ, π, charged) in the entire range of ^13^P CSs (Figure 2).

**Figure 3:**
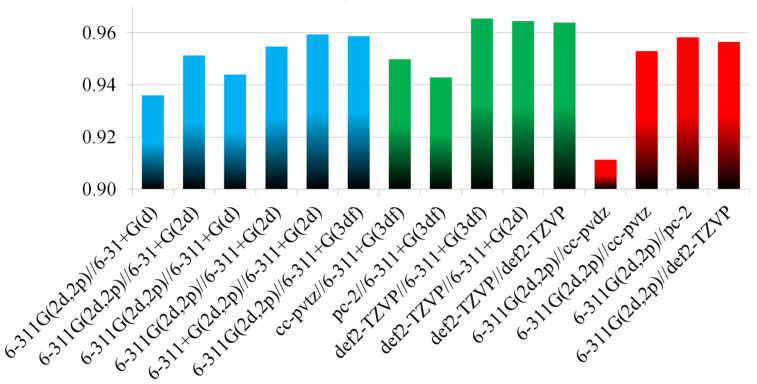
*R*^2^ dependence for the “training” set of Pd-complexes on combinations used in calculations. In all cases, the functional was fixed to the PBE0.

It was previously shown that improving the quality of the basis set at the geometry optimization stage leads to significant improvement in the calculated ^13^C NMR shifts in nickel complexes [10]. Therefore, by analogy, the first step was an attempt to “enhance” the basis functions within the Pople’s style basis sets. Indeed, the addition of one more polarization function for heavy elements leads to an increase in the correlation coefficient between calculated and experimental CSs (Table 1, Figure 3). The use of a basis set of triple-ζ quality also improves the *R*^2^ (Table 1, Figure 3). The interplay of these modifications on the basis sets leads to the best results (Table 1, Figure 3), and the systematic underestimation of the P=O CSs decreases by half (by ~5–15 ppm), although it cannot be completely corrected even when using this resource-intensive combination (Table S4).

On the other hand, the enlarging of the basis sets on the elements at the CS calculation stage by adding diffuse functions leads only to a minimal improvement, although the calculations become very time-consuming (Figure 3). Thus, further augmentation of the basis sets at the shielding calculation stage does not seem rational.

As a last resort, in an attempt to improve the results of calculations, we further augmented the basis sets at the optimization stage by adding another polarization function on the elements. Indeed, this led to some improvement in the agreement of the calculated CS with the experimental ones for problematic phosphorus atoms (Table 1, entry 6), although the time costs increased significantly (vide infra).

Thus, we concluded that the more flexible the basis set is on heavy elements at the geometry optimization stage, the better the structure is, and as a result, the better the ^31^P NMR shifts. However, the reason for the imperfect agreement between the calculation and experiment may also be in the quality of the basis sets on the metal. For example, for nickel complexes, diffusion functions on the metal are important for obtaining the correct geometry of the complex and, accordingly, its ^13^C NMR shifts [10]. Unfortunately, in the case of palladium, the choice of basis sets is very limited. Of the currently available the def2-TZVPD ECP with diffuse functions on valence orbitals is the most extended basis set defined [21,29]. Thus, calculations were carried out using this ECP on palladium and 6-311G(2d,2p)//6-311+G(2d) basis sets on the elements at the shielding//optimization calculation stages, respectively. However, it was found that, in general, the use of this ECP does not lead to an improvement, compared with the comb_1 combination with SDD ECP on Pd (Table 1, entry 7).

A rare alternative to ECP on palladium is the Sapporo-DKH3-DZP all-electron (AE) basis set [30,31], which is a compact Gaussian-type basis set for relativistic molecular calculations. However, the use of this AE basis set on palladium leads even to worse results. For example, if to use the basis set for Pd at the geometry optimization stage (Table 1, entry 8) the systematic underestimation of the P=O CSs increases (20 versus 18 ppm). Moreover, an application of this basis set for Pd at both stages of calculations leads to the worst results. Specifically, in addition to P=O (∆*δ* = 28 ppm), the P=P (∆*δ* = 65 ppm) phosphorus also starts to deviate from the main correlation (Table 1, entry 9). Thus, it appears that this basis set for Pd is not good for NMR shielding calculations.

Up to this point, we had checked only Pople’s basis sets on elements. As an alternative, three more popular basis sets, which are often used to calculate magnetic properties in organic systems, were tested. There are Dunning’s [32,33], Jensen’s [34,35,36], and Alrichs’s [29,37,38] basis sets.

First, we analyzed the performance of the basis set for the CS calculation stage using the best geometry optimized at the PBE0/6-311+G(3df) level. It was revealed that the use of Dunning’s correlation-consistent basis sets of triple-ζ quality (cc-pVTZ) for shielding calculation leads to some improvement, particularly for problematic P=O phosphorus (Table 1, entry 10). The Jensen’s polarization-consistent (pc-2) basis sets, which were specially developed for DFT calculations, showed worse results for P=O phosphorus (∆*δ* = 19 ppm). Additionally, the best results are observed if the Alrichs’s (def2-TZVP) basis sets were used for elements (∆*δ* = 9 ppm).

Next, we considered how these basis sets perform at the geometry optimization stage while the PBE0/6-311G(2d,2p) level was applied as a standard for CS calculation (Table 1, entry 16–19). We started with the cc-pVDZ (double-ζ) for optimization. The use of this basis set for geometry optimization leads to a very poor correlation between calculation and experiment (*R*^2^ = 0.911). Changing from the basis set of double- to triple-ζ quality (cc-pVTZ) leads to notable improvement, and the *R*^2^ becomes close to that obtained with PBE0/6-311+G(2d) geometry. The use of pc-2 or def2-TZVP basis sets results in ^31^P shifts for problematic phosphorus that are only slightly better than the corresponding Pople’s combination (comb_4), although the time costs increase substantially (vide infra).

Thus, the def2-TZVP//6-311+G(3df) combination looks the best. However, it is very difficult to work with in terms of both geometry optimization and CS calculation. Therefore, taking into account practical aspects, options with lighter basis sets were considered both at the geometry optimization stage and shielding calculation. It was found that, for example, the PBE0/6-311+G(2d) geometry with the def2-TZVP basis at the shielding calculation reproduces the experimental values quite well (∆*δ* = 13 ppm). At the same time, easing the basis set at the shielding calculation stage just to TZV leads to a significant deterioration in the results (∆*δ* = 81 ppm).

Several other combinations were also tested (Table 1, entries 20–22). Of those considered, only the use of the def2-TZVP basis at both stages of the calculation leads to a good correlation between the calculation and experiment (*R*^2^ = 0.964). The rest of the combinations do not look promising. Moreover, some variants proved to be bad, although, for example, in the case of ^13^C shifts in nickel complexes, they performed exceptionally well [10]. It should be noted that, in this case, both the P=O and phosphorus for systems with multiple bonds begin to deviate strongly from the general dependence (Table 1, entry 20).

Thus, based on the analysis performed on the training set, we came to the conclusion that several combinations are promising, leading to approximately the same correlation coefficients between the calculation and experiments. However, on the other hand, these approximations vary greatly in the time spent on calculations. Therefore, taking into account these factors, some of them seem to be more preferable from a practical point of view.

### 2.3. Practical Aspects, Quality of Calculations, and Computational Costs

The aim of this study is not just to find an approach that will allow ^31^P shifts to be estimated in Pd complexes with sufficient accuracy but to develop a tool that can be relatively easily applied for large molecules of practical interest using modest computer resources. Unfortunately, not all the approximations discussed above satisfy these requirements. Therefore, in this section, we discuss a comparative analysis of computational costs in order to choose the leading combinations in terms of the “price–quality” ratio.

**Figure 4:**
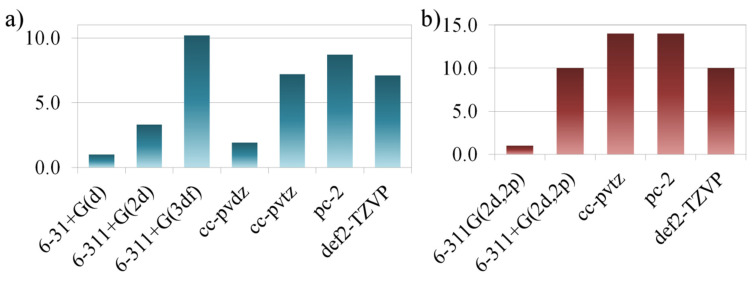
Relative time spent for 22 using different basis sets at the geometry optimization stage (**a**) and calculation of ^31^P shift (**b**).

First of all, let us consider the optimization stage since it is the most time-consuming. Complex **22** was taken as an example, and all optimizations were carried out from the same starting geometry. If we take the PBE0/6-31+G(d) approximation as a reference for time costs, then among the Pople’s type basis sets, geometry optimization using the 6-311+G(2d) and 6-311+G(3df) basis sets takes ~3 and ~10 times longer, respectively (Figure 4a). For the geometry optimization stage, this is essential, since, when performing calculations on a multiprocessor system, for example, it may take several days.

In turn, using Dunning’s basis sets of double- or triple-ζ quality is ~2 and ~7 times longer than using a 6-31+G(d) basis. Finally, the Jensen’s and Alrichs’s basis sets are ~7–9 times more time-consuming than the 6-31+G(d) basis set. Thus, at the geometry optimization stage, these basis sets are several times more time-consuming than the Pople’s 6-311+G(2d) basis sets at close values of *R*^2^ (Figure 3).

As for the CS calculation stage, when adding diffuse functions to the starting basis 6-311G(2d,2p), the “time–cost” increases by an order of magnitude only with a slight increase in quality (Figure 4b). Moreover, calculations using Dunning’s and Jensen’s basis sets of triple-ζ quality are lengthened by ~14 times, compared with 6-311G(2d,2p) at lower quality. Using the Alrichs’s basis set leads to an increase in time by about an order of magnitude, but it leads to a gain in quality, compared with the Pople’s basis set.

Thus, taking into account practical aspects, preference can be given to two combinations, in order to check their quality on a more extended test set—namely, the 6-311+G(2d) level looks optimal for the geometry optimization stage, while for the shielding calculation, the use of 6-311G(2d,2p) and def2-TZVP basis sets looks promising.

### 2.4. Checking the Leading Combinations on the Entire Test Set of Pd Complexes

**Figure 5:**
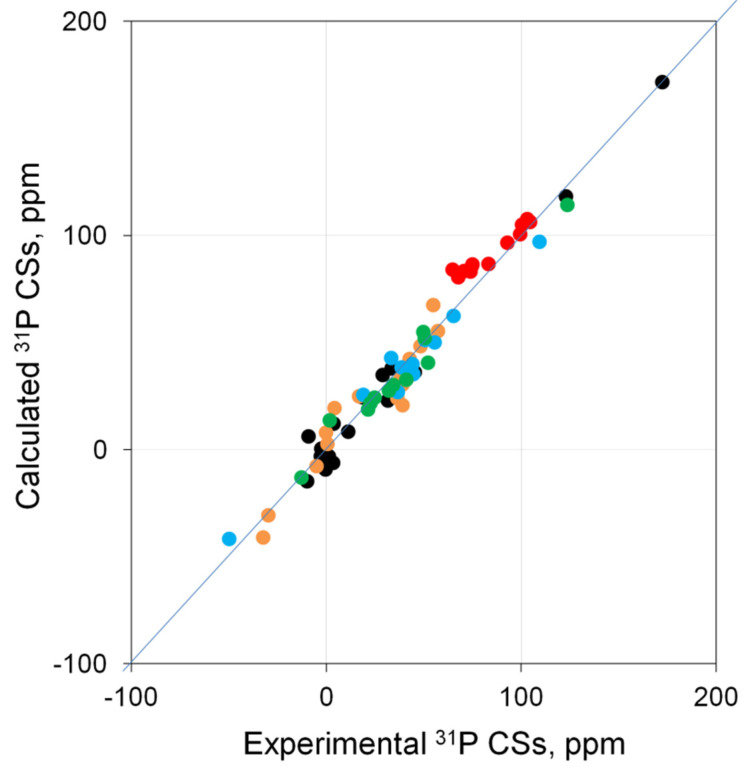
Correlation of calculated (PBE0/{6-311G(2d,2p); Pd(SDD)}//PBE0/{6-311+G(2d); Pd(SDD)}) vs. experimental ^31^P CSs for palladium complexes **1**–**39**. Notation of the complexes is the same as that in Figure 1.

The calculations performed for the entire test set using the PBE0/{6-311G(2d,2p); Pd(SDD)}//PBE0/{6-311+G(2d); Pd(SDD)} combination showed that improvement is observed for all problematic phosphorus (*R*^2^ = 0.963 versus 0.954), while for “normal” complexes, the changes are minimal (Figure 5). In other words, if there is no P=O phosphorus in the complex, then the lighter comb_1 combination is also sufficient.

At the same time, a more careful analysis reveals limitations typical for this level of calculations in the form of systematic errors when moving to lower fields. However, it is easy to minimize such errors by performing a linear scaling procedure [26,27,28] according to Equation (1) using the regression analysis parameters for the test set (Table 2).

*δ*_scaled_ = (*δ* − Intercept)/Slope, (1)

where *δ* is the calculated CS for a particular nucleus, and intercept and slope are scaling factors.

Thus, the application of this procedure allows for the evaluation of ^31^P shifts in palladium complexes with *RMSE* = 8.0 ppm.

Similar calculations using (PBE0/{def2-TZVP; Pd(SDD)}//PBE0/{6-311+G(2d); Pd(SDD)}) approximation for the entire test set lead to even higher correlation coefficients (*R*^2^ = 0.972). The linear scaling procedure allows for estimates of ^31^P CS with the best *RMSE* = 6.9 ppm (Figure S5).

### 2.5. Impact of Coordination Type on ^31^P NMR Shifts

The NMR shifts of the atoms directly involved in the formation of the complex with the TM strongly depend on the particular structure. Therefore, a comparison of calculated and experimental ^31^P NMR shifts can be used as a reliable tool to confirm or rule out hypothetical structures. The effectiveness of the tool can be demonstrated by the example of structure determination of complexes based on triorganylcyclotriphosphines [39]. In general, in such compounds, cyclophosphines can bind with TM in the *η*^2^ or *η*^1^,*η*^1^ fashions. According to X-ray data, in the case of Pd, the P=P double bond coordinates with transition metals in the *η*^2^ form, while for Pt, they undergo a ring-opening process during the reaction.

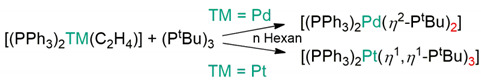



**Figure 6:**
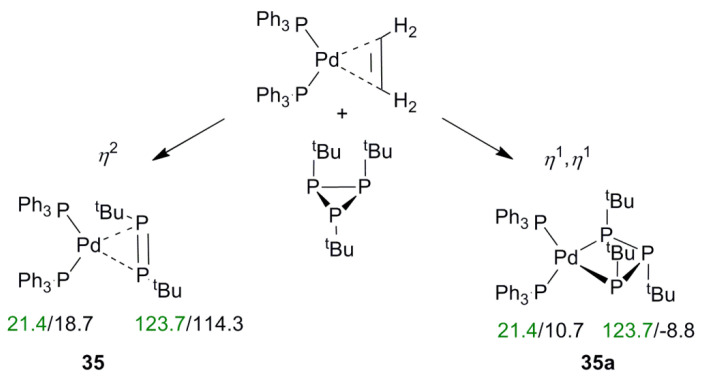
Structures of complexes **35** and **35a** with experimental (green) and calculated (black) ^31^P CSs (in ppm) included.

Indeed, according to calculations, for complex **35**, for example, both types correspond to an energy minimum (Figure 6). However, only for the *η*^2^ form (**35**, Figure 6) is the difference in the calculated (comb_3) versus the experimental ^31^P NMR shifts small (ca. 9 ppm), while for the *η*^1^,*η*^1^ form, with the ring-opening process of complex 35 (**a**), the deviation is more than 130 ppm (Figure 6). Thus, comparing theoretical results with those obtained by experiment is an easy way to determine its coordination type in solution as a π complex.

## 3. Materials and Methods

### Calculations

The quantum chemical calculations were carried out within the framework of the Kohn Sham density functional theory [13], with the Gaussian 03 [40] (Revision B.04) and the Gaussian 16 [41] (Revision A.03) software packages by using PBE0 [42] functional and a number of families of basis sets (Pople’s [43,44,45,46,47,48,49,50], Ahlrichs’s [29,37,38], Dunning’s [32,33], Jensen’s [34,35,36] sets). For the Pd center, the quasi-relativistic Stuttgart–Dresden ECP28MWB was used with corresponding (8*s*7*p*6*d*)/(6*s*5*p*3*d*) GTO valence basis set [24] (denoted as “(SDD)”), Sapporo-DKH3-DZP-diffuse (deprecated) basis set [30,31], and def2-TZVPD basis set [21,29]. Wherever possible, geometry optimization was started from an X-ray structure. For most of the complexes, the calculations were carried out for all possible conformers/isomers, and results for the lowest energy forms were used in the analysis. To take into account the medium effects, calculations were also carried out in the framework of the Polarizable Continuum Model [51] (denoted as “PCM”), with chloroform as solvent. To determine the energies of triplet states, geometry optimizations were performed at the spin-unrestricted formalism (UPBE0/6-31+G(d)). ^31^P NMR CSs were calculated by the GIAO method [25]. All ^31^P data were referenced to H_3_PO_4_.

The pc-2, def2-TZVP, def2-TZVPD, and Sapporo-DKH3-DZP-diffuse basis sets were downloaded from the EMSL basis set library for the Gaussian package [52].

The Gaussian 03 calculations were carried out on a PC with IntelCore i7-3970X CPU, 3.5 GHz. Calculations with 6-311+G(3df), pc-2, cc-pVTZ, def2-TZVP, and Sapporo-DKH3-DZP-diffuse (deprecated) basis sets were carried out with the Gaussian 16 on 20 CPUs, IntelXeon ES-2650 2.20 GHz.

## 4. Conclusions

Comparative analysis of calculated (GIAO method, DFT level) and experimental ^31^P NMR shifts for a wide range of model palladium complexes showed that, on the whole, the theory reproduces the experimental data well. The exceptions are the complexes with P=O phosphorus, for which there is a systematic underestimation of shielding, the value of which depends on the flexibility of the basis sets, especially at the geometry optimization stage. The use of basis sets of triple-ζ quality and additional polarization functions at this stage reduces the underestimation of shielding for such phosphorus compounds.

To summarize, in practice, for the rapid assessment of phosphorus CS, with the exception of the P=O type, a rather simple PBE0/{6-311G(2d,2p); Pd(SDD)}//PBE0/{6-31+G(d); Pd(SDD)} approximation is quite acceptable (*RMSE* = 8.9 ppm). Optimal from the point of view of “price-quality” ratio is the PBE0/{6-311G(2d,2p); Pd(SDD)}//PBE0/{6-311+G(2d); Pd(SDD)} (*RMSE* = 8.0 ppm) and the PBE0/{def2-TZVP; Pd(SDD)}//PBE0/{6-311+G(2d); Pd(SDD)} (*RMSE* = 6.9 ppm) approaches. The use of the PBE0/6-311+G(3df) level at the geometry optimization stage leads to some further improvement in the calculation, but the requirements for computer resources are increasing dramatically. In all cases, a linear scaling procedure is necessary to minimize systematic errors.

## Figures and Tables

**Table 1:** *R*^2^ dependence on combination used in the calculations for the “training” set of Pd complexes (**2**, **9**, **17**, **22**–**23**, **26**, **28**–**30**, **32**, **38**).

Entry	Combination	Basis Set *^a^*		*R* ^2^	Δ*δ* *^b^*, ppm
		Elements	Pd		
1	comb_1	6-311G(2d,2p)//6-31+G(d)	SDD//SDD	0.936	24
2	comb_2	6-311G(2d,2p)//6-31+G(2d)	SDD//SDD	0.951	19
3	comb_3	6-311G(2d,2p)//6-311+G(d)	SDD//SDD	0.944	22
4	comb_4	6-311G(2d,2p)//6-311+G(2d)	SDD//SDD	0.955	18
5	comb_5	6-311+G(2d,2p)//6-311+G(2d)	SDD//SDD	0.959	18
6	comb_6	6-311G(2d,2p)//6-311+G(3df)	SDD//SDD	0.959	14
7	comb_7	6-311G(2d,2p)//6-311+G(2d)	def2-TZVPD//def2-TZVPD	0.953	19
8	comb_8	6-311G(2d,2p)//6-311+G(2d)	SDD//DKH3-DZP	0.904	20
9	comb_9	6-311G(2d,2p)//6-311+G(2d)	DKH3-DZP//DKH3-DZP	0.829	28 (65) *^c^*
10	comb_10	cc-pVTZ//6-311+G(3df)	SDD//SDD	0.950	15
11	comb_11	pc-2//6-311+G(3df)	SDD//SDD	0.943	19
12	comb_12	def2-TZVP//6-311+G(3df)	SDD//SDD	0.965	9
13	comb_13	def2-TZVP//6-311+G(2d)	SDD//SDD	0.964	13
14	comb_14	TZV//6-311+G(3df)	SDD//SDD	0.833	68
15	comb_15	def2-TZVP//def2-TZVP	SDD//SDD	0.964	11
16	comb_16	6-311G(2d,2p)//cc-pVDZ	SDD//SDD	0.911	36
17	comb_17	6-311G(2d,2p)//cc-pVTZ	SDD//SDD	0.953	17
18	comb_18	6-311G(2d,2p)//pc-2	SDD//SDD	0.958	11
19	comb_19	6-311G(2d,2p)//def2-TZVP	SDD//SDD	0.956	15
20	comb_20	TZV//TZV	SDD//SDD	0.689	118 (101) *^c^*
21	comb_21	cc-pVTZ//6-31+G(d)	SDD//SDD	0.927	22
22	comb_22	cc-pVTZ//cc-pVDZ	SDD//SDD	0.901	37

*^a^* Basis sets used for shielding calculation//geometry optimization steps; *^b^* average deviation from the general correlation line for P=O phosphorus atoms; *^c^* in brackets average deviation from the general correlation line for P=P phosphorus atoms.

**Table 2:** Empirical scaling factors obtained by the linear regression analysis of calculated and experimental δ^31^P for the title Pd complexes.

Level of Theory	*R* ^2^	Slope	Intercept	*RMSE*
PBE0/6-311G(2d,2p)//PBE0/6-31+G(d)	0.954	1.1766	−12.297	8.9
PBE0/6-311G(2d,2p)//PBE0/6-311+G(2d)	0.963	1.1346	−13.8962	8.0
PBE0/def2-TZVP//PBE0/6-311+G(2d)	0.972	0.9297	1.1235	6.9

## Data Availability

All data are contained within the article and Supporting Information.

## Supplementary Materials

The following supporting information can be downloaded at: https://www.mdpi.com/article/10.3390/molecules27092668/s1, Structures of all model complexes [53,54,55,56,57,58,59,60,61,62,63,64,65,66,67,68,69,70] with experimental ^31^P NMR shifts and all calculation results. Table S1: Experimental and calculated ^31^P NMR shifts (ppm) for all model complexes. Table S2: Empirical scaling factors obtained by the linear regression analysis of calculated and experimental *δ*^31^P for the title Pd-complexes. Table S3: Triplet-singlet states energy gap (UPBE0/6-31+G(d)) for the “training” set of Pd complexes (**2**, **9**, **17**, **22**–**23**, **26**, **28**–**30**, **32**, **38**). Table S4: Experimental and calculated (by different combinations) ^31^P NMR shifts (ppm) for the “training” set of Pd complexes (**2**, **9**, **17**, **22**–**23**, **26**, **28**–**30**, **32**, **38**).

## Sample Availability

Samples of the compounds are not available from the authors.
