# Supplementary material for: DFT Calculations of 31P NMR Chemical Shifts in Palladium Complexes
Source: Molecules. 2022 Apr 21;27(9):2668. doi: 10.3390/molecules27092668 (PMC9105066; doi:10.3390/molecules27092668)
Supplement: Supplementary file 1 [file molecules-27-02668-s001.zip › molecules-1658311-supplementary.pdf]

Supplementary Information for:

DFT Calculations of ^{31}P NMR Chemical Shifts in Palladium Complexes

*Svetlana A. Kondrashova, Fedor M. Polyancev, Shamil K. Latypov**

Arbuzov Institute of Organic and Physical Chemistry, FRC Kazan Scientific Center of RAS,
Kazan, Tatarstan 420088, Russian Federation

TABLE OF CONTENTS **pages**

CALCULATIONAL DETAILS 3

Figure S1. Complexes based on σ-donor ligands. 4

Figure S2. Charged complexes based on σ-donor ligands. 4

Figure S3. Complexes containing P=O groups. 5

Figure S4. Complexes based on π-donating ligands. 5

Table S1. Experimental and calculated ^{31}P NMR shifts (ppm) for all model complexes. 6

Table S2. Empirical scaling factors obtained by the linear regression analysis of calculated and experimental δ^{31}P for the title Pd-complexes. 9

Table S3. Triplet-singlet states energy gap (UPBE0/6-31+G(d)) for the "training" set of Pd complexes (**2, 9, 17, 22-23, 26, 28-30, 32, 38**). 10

Table S4. Experimental and calculated (by different combinations) ^{31}P NMR shifts (ppm) for the "training" set of Pd complexes (**2, 9, 17, 22-23, 26, 28-30, 32, 38**). ... 11

Figure S5. Correlation of calculated (PBE0/{def2-TZVP; Pd(SDD)}//PBE0/{6-311+G(2d); Pd(SDD)}, scaled) *vs* experimental ^{31}P CSs for palladium complexes **1-39**. Complexes based on σ-donor ligands (●), charged palladium complexes based on σ-donor ligands (●), complexes containing P=O groups (● are the P = O phosphorus, ● are other phosphorus atoms in this type of complexes) and complexes based on π-donor ligands (●). 13

REFERENCES 14

CALCULATIONAL DETAILS

The quantum chemical calculations have been carried out within the framework of the Kohn Sham density functional theory [1] with the Gaussian 03 [2] (Revision B.04) and the Gaussian 16 [3] (Revision A.03) software packages by using PBE0 [4] functional and a number of families of basis sets (Pople's [5-12], Ahlrichs's [13-15], Dunning's [16-17], Jensen's [18-20]). For the Pd center was used the quasi-relativistic Stuttgart-Dresden ECP28MWB with corresponding (8*s*7*p*6*d*)/[6*s*5*p*3*d*] GTO valence basis set [21] (denoted as "(SDD)"), Sappo-ro-DKH3-DZP-diffuse [deprecated] basis set [22-23] and def2-TZVPD basis set [24-25]. Wherever possible, geometry optimization was started from X-ray structure. For most of the complexes the calculations were carried out for all possible conformers/isomers and results for the lowest energy forms were used in analysis. To take into account the medium effects, calculations were also carried out in the framework of Polarizable Contin-uum Model [26] (denoted as "PCM") with chloroform as solvent. To determine the energies of triplet states geometry optimizations were performed at the spin-unrestricted formalism (UPBE0/6-31+G(d)). ^{31}P NMR CSs were calculated by the GIAO method [27]. All ^{31}P data was referenced to H_3PO_4.

The pc-2, def2-TZVP, def2-TZVPD and Sapporo-DKH3-DZP-diffuse basis sets were downloaded from the EMSL basis set library for Gaussian package [28].

The Gaussian 03 calculations were carried out on a PC with IntelCore i7-3970X CPU, 3.5 GHz. Calculations with 6-311+G(3df), pc-2, cc-pVTZ, def2-TZVP, and Sappo-ro-DKH3-DZP-diffuse [deprecated] basis sets were carried out with the Gaussian 16 on 20 CPUs, IntelXeon ES-2650 2.20 GHz.

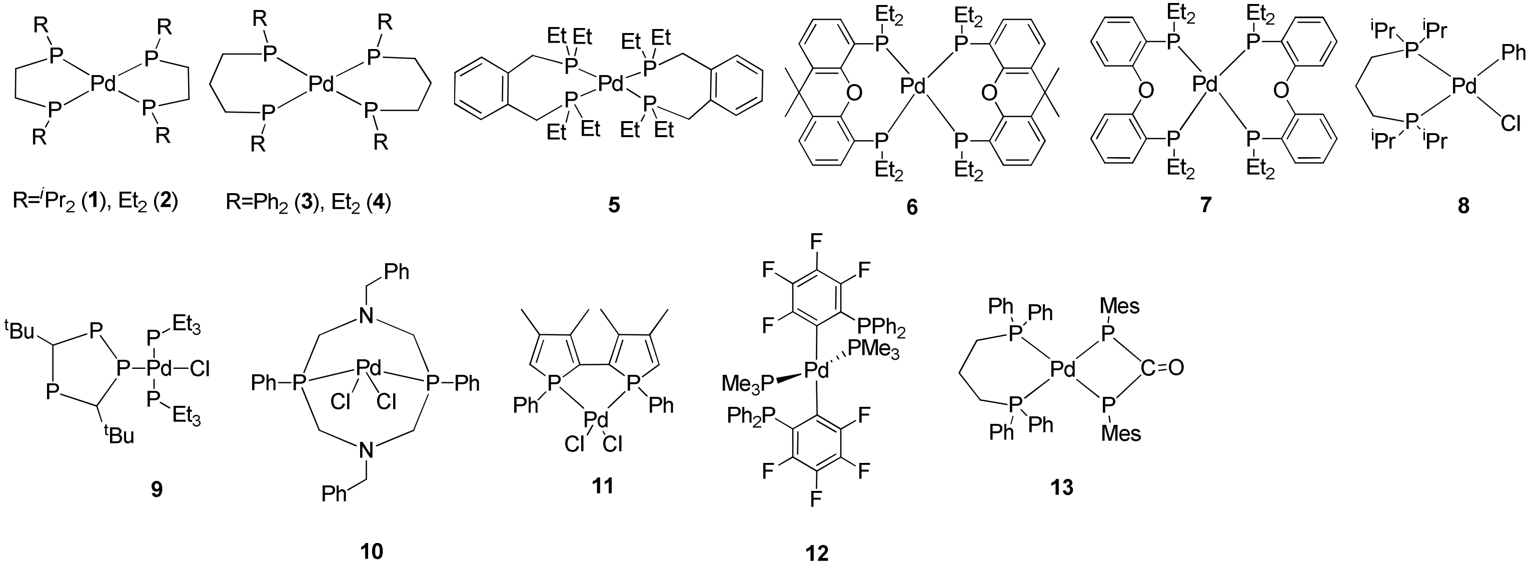

Figure S1. Complexes based on σ-donor ligands.

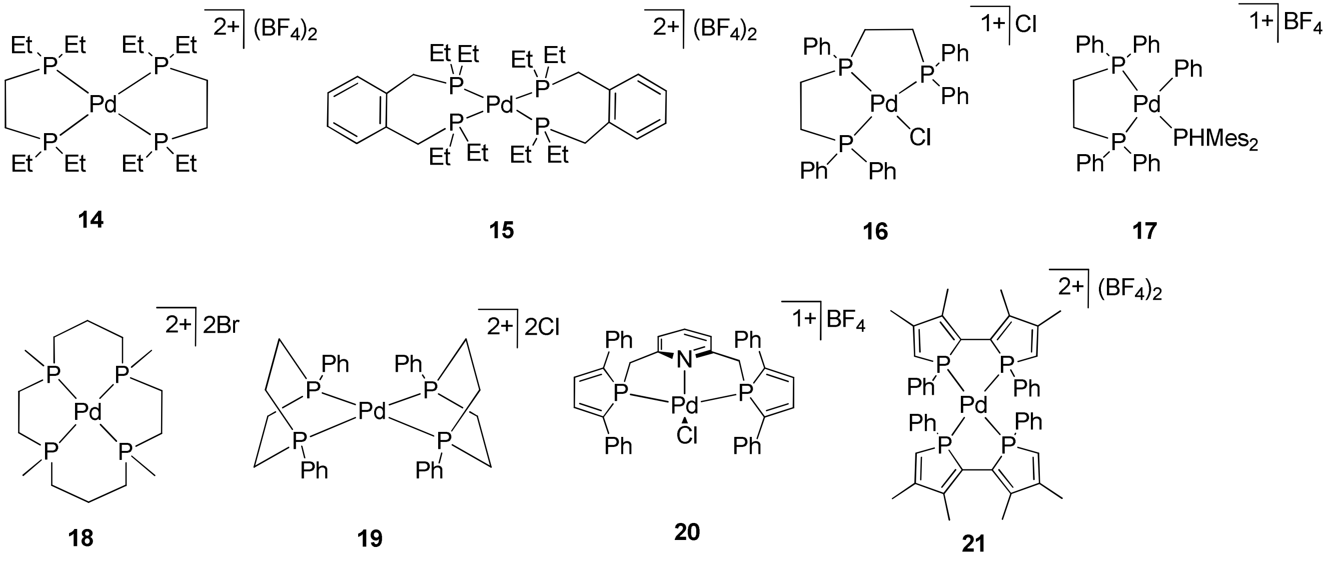

Figure S2. Charged complexes based on σ-donor ligands.

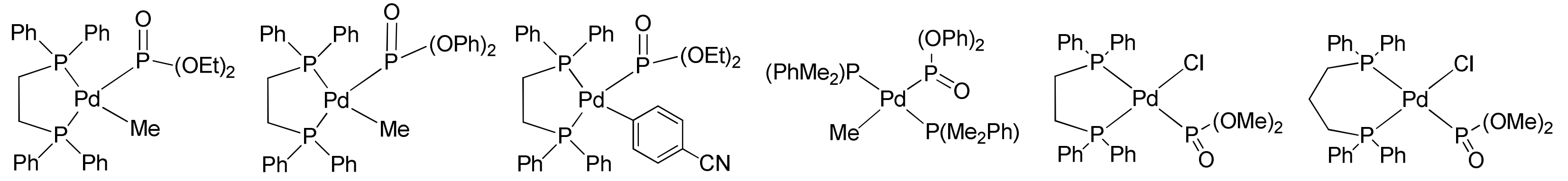

22 **23** **24** **25** **26** **27**

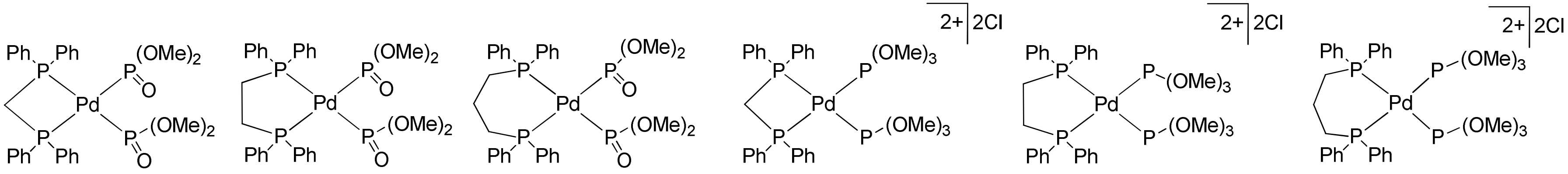

28 **29** **30** **31** **32** **33**

Figure S3. Complexes containing P=O groups.

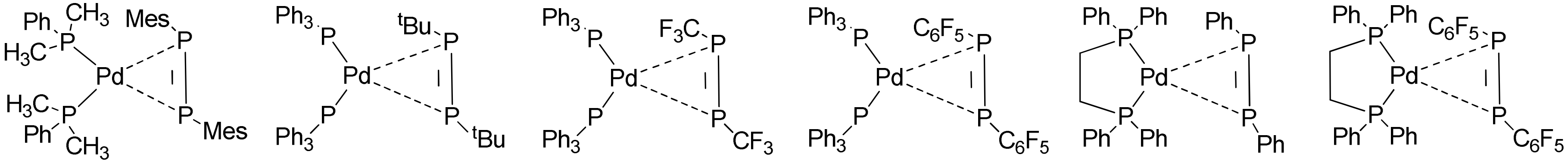

34 **35** **36** **37** **38** **39**

Figure S4. Complexes based on π-donating ligands.

Table S1. Experimental and calculated ^{31}P NMR shifts (ppm) for all model complexes.

		Calculated								
Compound	Experiment	PBE0/6-311G(2d,2p)// PBE0/6-31+G(d)	PBE0/6-311G(2d,2p)// PBE0/6-31+G(d)[a]	PBE0/6-311G(2d,2p) (PCM)// PBE0/6-31+G(d) (PCM)	PBE0/6-311G(2d,2p) (PCM)// PBE0/6-31+G(d) (PCM)[a]	PBE0/6-311G(2d,2p)// PBE0/6-311+G(2d)	PBE0/6-311G(2d,2p)// PBE0/6-311+G(2d)[a]	PBE0/def2-TZVP// PBE0/6-311+G(2d)	PBE0/ def2-TZVP // PBE0/6-311+G(2d)[a]	Reference
**1**	45.5	22.5	29.6	29.7	33.9	27.1	36.1	43.7	38.1	29
**2**	31.5	15.0	23.2	14.2	20.8	12.0	22.8	28.1	24.2	30
**3**	3.7	-2.0	8.8	-3.0	6.4	-0.1	12.2	12.3	10.1	29
**4**	-2.6	-14.0	-1.4	-14.2	-3.1	-13.3	0.5	-2.2	-2.8	30
**5**	1.3	-17.6	-4.5	-14.4	-3.2	-17.5	-3.2	-1.4	-2.1	30
	-0.4	-18.1	-4.9	-21.5	-9.2	-24.5	-9.3	-9.0	-8.8	
**6**	-0.9	-19.1	-5.8	-18.6	-6.8	-21.9	-7.1	-5.6	-5.8	30
	-9.9	-27.6	-13.0	-27.3	-14.1	-30.7	-14.8	-13.1	-12.5	
**7**	-1.3	-20.1	-6.6	-19.1	-7.2	-23.3	-8.3	-8.4	-8.3	30
**8**	11.2	-6.3	5.1	-3.2	6.2	-4.4	8.4	6.1	4.6	29
	29.0	23.1	30.1	30.9	34.9	25.5	34.7	36.0	31.2	
**9**	123.0	121.3	113.5	143.0	129.2	120.2	118.2	133.9	118.4	31
	18.5	17.0	24.9	18.1	24.1	13.8	24.4	30.0	25.9	
**10**	-9.2	-9.4	2.5	-7.5	2.6	-6.9	6.2	3.7	2.5	32
**11**	33.4	30.7	36.5	36.9	39.9	29.1	37.9	42.0	36.6	33
**12**	-2.9	-17.1	-4.1	-18.0	-6.3	-17.4	-3.1	-8.4	-8.3	34
**13**	3.5	-17.7	-4.6	-14.9	-3.7	-20.9	-6.2	2.0	1.0	35
	172.3	183.3	166.2	166.7	149.2	180.7	171.5	200.0	177.2	
**14**	65.2	60.6	62.0	60.2	59.6	56.8	62.3	72.1	63.3	30
**15**	19.0	15.2	23.4	14.9	21.4	15.0	25.5	26.5	22.8	30
**16**	44.2	34.1	39.4	39.6	42.2	31.3	39.8	47.8	41.7	36
	109.3	99.2	94.8	109.5	101.1	96.3	97.1	114.0	100.6	

**17**	50.6	38.0	42.7	48.1	49.4	44.1	51.1	61.8	54.2	37
	44.6	26.4	32.9	30.1	34.2	26.1	35.3	41.4	36.0	
	-49.9	-58.2	-39.0	-60.2	-41.8	-61.2	-41.7	-48.6	-44.1	
**18**	38.5	33.6	39.0	33.5	37.1	29.5	38.2	43.8	38.2	38
**19**	55.6	46.2	49.7	47.8	49.1	42.8	50.0	58.2	51.0	39
**20**	33.2	25.1	31.8	35.5	38.8	34.6	42.7	47.0	41.0	40
**21**	36.6	29.4	35.4	28.1	32.5	16.6	26.9	40.0	34.8	33
**22**	83.1	95.2	91.4	103.2	95.8	84.5	86.7	96.0	84.6	41
	36.5	14.9	23.1	20.0	25.7	13.4	24.1	31.2	26.9	
	42.6	37.0	41.9	41.5	43.8	34.2	42.4	49.9	43.6	
**23**	92.9	101.9	97.1	116.4	106.9	95.8	96.7	107.0	94.4	42
	48.3	44.6	48.4	49.0	50.1	40.9	48.3	56.4	49.4	
	40.1	29.8	35.8	30.4	34.5	24.3	33.7	40.1	34.9	
**24**	74.7	106.5	101.0	99.6	92.7	83.4	85.8	94.5	83.3	41
	38.0	23.9	30.8	30.6	34.6	23.7	33.1	37.1	32.2	
	39.0	36.3	41.3	36.0	39.2	20.7	30.5	40.8	35.5	
**25**	99.5	111.5	105.2	115.4	106.0	100.1	100.5	110.1	97.2	42
	-0.2	-1.3	9.3	-0.9	8.1	-4.8	8.0	10.6	8.6	
**26**	39.2	13.6	22.0	24.1	29.2	9.5	20.6	24.9	21.3	43
	57.3	50.9	53.7	62.7	61.7	48.8	55.3	65.3	57.3	
	67.7	87.3	84.6	91.5	85.9	77.4	80.5	88.2	77.7	
**27**	-5.1	-19.9	-6.5	-12.2	-1.4	-22.7	-7.8	-7.1	-7.1	43
	16.7	16.7	24.6	23.5	28.7	14.4	24.9	30.6	26.4	
	64.8	90.4	87.3	95.5	89.3	81.3	83.9	91.8	80.9	
**28**	-29.7	-47.6	-30.0	-46.8	-30.5	-48.8	-30.8	-32.5	-29.8	43
	75.0	94.2	90.5	99.1	92.3	84.3	86.5	94.2	83.0	
**29**	43.1	22.9	29.9	38.6	41.4	25.7	34.9	43.2	37.6	43
	73.9	85.2	82.9	96.5	90.1	80.3	83.0	90.3	79.5	
**30**	0.5	-8.9	2.9	-4.7	4.9	-11.0	2.6	4.6	3.3	43
	70.5	89.7	86.7	94.9	88.8	80.6	83.3	91.2	80.3	
**31**	-32.4	-58.7	-39.4	-56.2	-38.4	-60.4	-41.0	-45.8	-41.6	43
	104.5	114.7	107.9	115.8	106.4	106.6	106.2	117.5	103.8	
**32**	54.9	66.9	67.3	64.9	63.5	62.7	67.5	78.4	69.0	43
	103.0	112.7	106.2	117.1	107.4	108.2	107.6	118.8	104.9	
**33**	4.2	10.7	19.5	11.5	18.6	8.1	19.4	24.1	20.6	43
	100.3	113.0	106.5	113.7	104.6	105.2	105.0	116.1	102.5	
**34**	49.7	53.1	55.6	38.2	41.0	48.3	54.8	62.2	54.5	44

	-12.8	-26.1	-11.7	-23.6	-11.0	-28.7	-13.0	-13.6	-12.9	
**35**	123.7	121.0	113.3	106.8	98.8	115.8	114.3	128.4	113.5	45
	21.4	9.0	18.1	9.8	17.1	7.3	18.7	22.9	19.6	
**36**	50.5	49.0	52.1	35.6	38.9	45.1	52.0	57.4	50.3	46
	22.7	12.4	21.0	13.6	20.3	10.7	21.7	25.1	21.5	
**37**	32.0	22.1	29.2	9.3	16.7	17.3	27.5	30.7	26.5	47
	24.8	15.2	23.4	15.9	22.3	13.7	24.3	29.3	25.3	
**38**	34.3	20.5	27.9	31.5	35.4	20.4	30.2	34.6	30.0	47
	41.0	25.9	32.5	27.3	31.9	23.1	32.6	39.2	34.1	
**39**	1.7	-4.0	7.1	-24.5	-11.7	1.5	13.6	14.4	12.0	47
	52.1	33.4	38.8	36.6	39.7	32.1	40.5	47.9	41.8	
R^2		**0.9535**	**0.9535**	**0.9447**	**0.9447**	**0.9627**	**0.9627**	**0.9720**	**0.9720**	
RMSE		**13.8**	**8.9**	**14.2**	**9.8**	**13.6**	**8.0**	**10.8**	**6.9**	

[a] corrected by using the factors for empirical linear scaling obtained by the linear regression procedure for the title Pd-complexes.

Table S2. Empirical scaling factors obtained by the linear regression analysis of calculated and experimental δ^{31}P for the title Pd-complexes.

Level of theory	R^2	Slope	Intercept	*RMSE*
PBE0/6-311G(2d,2p)//PBE0/6-31+G(d)	0.954	1.1766	-12.297	8.9
PBE0/6-311G(2d,2p) (PCM)//PBE0/6-31+G(d) (PCM)	0.945	1.1881	-10.561	9.8
PBE0/6-311G(2d,2p)//PBE0/6-311+G(2d)	0.963	1.1346	-13.8962	8.0
PBE0/def2-TZVP//PBE0/6-311+G(2d)	0.972	0.9297	1.1235	6.9

Table S3. Triplet-singlet states energy gap (UPBE0/6-31+G(d)) for the "training" set of Pd complexes (**2, 9, 17, 22-23, 26, 28-30, 32, 38**).

Compound	E, kcal/mol
**38**	38.9
**9**	38.9
**17**	46.6
**2**	51.7
**22**	46.1
**23**	44.9
**26**	40.9
**28**	-[a]
**29**	35.8
**30**	35.6
**32**	67.6

[a] triplet state does not correspond to energy minima.

Table S4. Experimental and calculated (by different combinations) ^{31}P NMR shifts (ppm) for the "training" set of Pd complexes (**2, 9, 17, 22-23, 26, 28-30, 32, 38**).

		Calculated																					
		Combination																					
Compound	Experimental	6-311G(2d,2p)// 6-31+G(d)	6-311G(2d,2p)// 6-31+G(2d)	6-311G(2d,2p)// 6-311+G(d)	6-311G(2d,2p)// 6-311+G(2d)	6-311+G(2d,2p)// 6-311+G(2d)	6-311G(2d,2p)// 6-311+G(3df)	6-311G(2d,2p)//6-311+G(2d) Pd - def2-TZVPD//def2-TZVPD	6-311G(2d,2p)//6-311+G(2d) Pd - SDD//DKH3-DZP	6-311G(2d,2p)//6-311+G(2d) Pd - DKH3-DZP//DKH3-DZP	cc-pVTZ// 6-311+G(3df)	pc-2// 6-311+G(3df)	def2-TZVP// 6-311+G(3df)	def2-TZVP// 6-311+G(2d)	TZV// 6-311+G(3df)	def2-TZVP// def2-TZVP	6-311G(2d,2p)// cc-pVDZ	6-311G(2d,2p)// cc-pVTZ	6-311G(2d,2p)// pc-2	6-311G(2d,2p)// def2-TZVP	TZV// TZV	cc-pVTZ// 6-31+G(d)	cc-pVTZ// cc-pVDZ
**2**	31.5	15.0	22.3	25.8	12.0	21.8	25.3	14.8	1.3	-4.0	13.7	27.2	31.3	28.1	-37.8	30.3	12.4	22.2	25.0	24.2	-58.2	3.2	-3.9
**9**	123.0	121.3	129.4	133.5	120.2	131.8	132.0	124.6	111.7	112.3	123.1	131.9	135.2	133.9	75.4	133.8	119.2	128.9	138.0	130.5	68.9	112.5	109.0
	18.5	17.0	24.3	27.8	13.8	24.0	25.8	17.6	22.8	21.7	7.4	33.4	31.9	30.0	-46.8	31.0	14.4	23.5	25.4	24.9	-66.4	-1.5	-6.9
**17**	50.6	38.0	53.4	57.8	44.1	56.2	56.4	47.9	49.1	47.2	37.3	64.2	64.2	61.8	-8.9	62.9	44.7	54.2	55.8	55.2	-32.8	28.5	22.6
	44.6	26.4	35.4	39.8	26.1	36.7	38.4	29.3	28.2	27.9	27.5	41.3	43.7	41.4	-33.9	42.6	25.7	35.8	37.7	37.3	-58.0	17.6	10.8
	-49.9	-58.2	-52.0	-47.3	-61.2	-51.7	-49.0	-57.6	-62.8	-68.3	-52.2	-44.7	-46.6	-48.6	-122.8	-48.6	-63.6	-53.6	-50.1	-51.0	-150.8	-64.2	-71.3
**38**	34.3	20.5	31.5	34.4	20.4	33.0	24.8	25.2	49.0	92.4	23.0	28.0	28.5	34.6	-33.4	28.7	22.6	25.9	27.0	24.6	52.4	11.4	15.7
	41.0	25.9	32.0	36.6	23.1	34.8	35.9	26.3	17.8	13.4	26.0	39.1	41.9	39.2	-0.8	40.9	23.3	33.5	35.2	34.6	-55.4	38.7	8.5
**22**	83.1[a]	95.2	96.7	104.4	84.5	94.4	93.7	88.9	90.0	93.2	85.4	92.2	95.0	96.0	76.7	94.6	104.7	95.9	94.3	93.4	119.8	85.6	89.1
	36.5	14.9	23.0	26.2	13.4	25.6	26.3	16.8	12.0	9.5	13.4	28.1	34.1	31.2	-39.5	32.8	12.3	23.3	25.3	24.9	-40.2	2.5	-3.9
	42.6	37.0	43.6	48.4	34.2	46.0	46.6	37.9	34.3	32.0	34.3	49.4	52.3	49.9	-19.9	51.3	36.1	44.5	46.0	45.5	-66.0	25.1	19.9
**23**	92.9	101.9	106.3	117.4	95.8	106.9	104.5	99.5	100.6	105.6	96.8	120.5	105.9	107.0	94.7	104.4	119.4	106.6	100.9	102.9	150.3	98.1	104.9
	48.3	44.6	50.6	55.2	40.9	52.9	53.2	44.4	38.5	21.9	41.9	72.9	58.7	56.4	-16.5	58.3	42.1	51.3	50.0	52.3	-37.7	33.3	29.1
	40.1	29.8	32.4	38.0	24.3	34.1	36.4	27.6	23.0	35.6	24.6	55.6	42.1	40.1	-32.7	40.6	23.4	33.7	32.5	34.7	-52.6	14.6	6.4
**26**	39.2	13.6	20.2	23.6	9.5	21.3	21.1	13.1	11.8	12.2	10.8	21.5	26.4	24.9	-45.8	26.3	13.4	20.7	21.7	20.6	-53.9	1.4	-5.0
	57.3	50.9	58.2	63.0	48.8	59.9	60.9	53.1	51.0	47.1	47.2	62.3	67.3	65.3	-14.4	66.3	47.8	58.4	60.2	59.8	-36.3	38.2	31.3
	67.7	87.3	89.6	95.6	77.4	87.0	86.1	81.4	83.0	85.4	78.1	85.5	87.1	88.2	77.1	87.4	96.2	89.1	87.5	86.4	115.0	79.1	82.6
**28**	-29.7	-47.6	-38.5	-35.9	-48.8	-36.6	-36.7	-45.1	-41.6	-50.4	-45.8	-34.2	-30.7	-32.5	-101.8	-31.6	-54.3	-40.2	-37.7	-37.9	-133.1	-55.1	-64.8
	75.0	94.2	95.3	103.2	84.3	93.5	93.2	88.7	88.5	90.6	84.9	91.9	93.7	94.2	80.8	93.6	106.0	95.5	93.9	93.0	117.4	85.2	92.1
**29**	43.1	22.9	35.3	39.4	25.7	38.4	37.9	29.0	20.5	11.5	21.8	43.0	45.6	43.2	-25.1	39.8	25.3	34.7	36.4	34.1	-48.7	15.6	8.1

	73.9	85.2	91.8	99.0	80.3	89.8	89.4	84.5	85.9	87.5	81.1	87.5	89.6	90.3	75.6	90.3	103.9	91.5	90.1	89.9	120.6	80.8	87.8
**30**	0.5	-8.9	-1.9	2.3	-11.0	-0.1	0.4	-7.8	-7.6	-11.5	-13.6	5.6	6.5	4.6	-67.3	5.5	-12.1	-2.3	0.0	-0.8	-94.8	-22.8	-30.0
	70.5	89.7	92.1	99.8	80.6	91.8	89.9	85.2	86.8	87.1	81.5	88.7	90.3	91.2	80.6	90.3	98.9	92.2	90.7	89.9	120.3	81.1	84.4
**32**	54.9	66.9	72.1	77.1	62.7	72.9	73.9	66.5	74.0	78.3	58.8	77.7	79.6	78.4	8.3	78.7	65.6	73.0	73.9	73.0	-4.7	51.8	47.0
	103.0	112.7	119.1	124.5	108.2	118.9	116.4	112.1	115.0	116.3	103.9	113.6	117.0	118.8	89.4	116.5	124.0	117.7	116.6	115.9	112.4	102.7	105.4
	R^2	**0.9359**	**0.9512**	**0.9439**	**0.9547**	**0.9593**	**0.9586**	**0.9530**	**0.9042**	**0.8287**	**0.9498**	**0.9428**	**0.9654**	**0.9644**	**0.8333**	**0.9638**	**0.9113**	**0.9529**	**0.9582**	**0.9564**	**0.6889**	**0.9271**	**0.9011**
	$\Delta\delta^b$	24	19	22	18	18	14	19	20	28 (65)[c]	15	19	9	13	68	11	36	17	11	15	118 (101)[d]	22	37

[a] ^{31}P NMR shifts of P=O phosphorus atoms colored in red; [b] average deviation (ppm) from the general correlation line for P=O phosphorus atoms; [c] in brackets average deviation from the general correlation line for P=P phosphorus atoms.

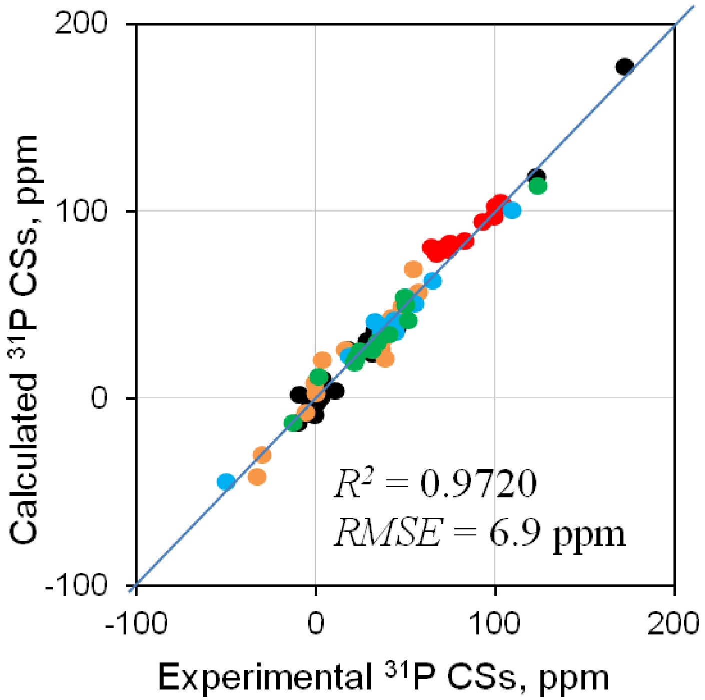

Figure S5. Correlation of calculated (PBE0/{def2-TZVP; Pd(SDD)}//PBE0/{6-311+G(2d); Pd(SDD)}, scaled) *vs* experimental ^{31}P CSs for palladium complexes **1-39**. Complexes based on σ-donor ligands (●), charged palladium complexes based on σ-donor ligands (●), complexes containing P=O groups (● are the P = O phosphorus, ● are other phosphorus atoms in this type of complexes) and complexes based on π-donor ligands (●).

REFERENCES

[1] Kohn, W.; Sham, L. J. Self-consistent equations including exchange and correlation effects. *Phys. Rev.*, **1965**, *140*, A1133.

[2] Frisch, M. J.; Trucks, G. W.; Schlegel, H. B.; Scuseria, G. E.; Robb, M. A.; Cheeseman, J. R.; Montgomery, J. A., Jr.; Vreven, T.; Kudin, K. N.; Burant, J. C.; Millam, J. M.; Iyengar, S. S.; Tomasi, J.; Barone, V.; Mennucci, B.; Cossi, M.; Scalmani, G.; Rega, N.; Petersson, G. A.; Nakatsuji, H.; Hada, M.; Ehara, M.; Toyota, K.; Fukuda, R.; Hasegawa, J.; Ishida, M.; Nakajima, T.; Honda, Y.; Kitao, O.; Nakai, H.; Klene, M.; Li, X.; Knox, J. E.; Hratchian, H. P.; Cross, J. B.; Adamo, C.; Jaramillo, J.; Gomperts, R.; Stratmann, R. E.; Yazyev, O.; Austin, A. J.; Cammi, R.; Pomelli, C.; Ochterski, J. W.; Ayala, P. Y.; Morokuma, K.; Voth, G. A.; Salvador, P.; Dannenberg, J. J.; Zakrzewski, V. G.; Dapprich, S.; Daniels, A. D.; Strain, M. C.; Farkas, O.; Malick, D. K.; Rabuck, A. D.; Raghavachari, K.; Foresman, J. B.; Ortiz, J. V.; Cui, Q.; Baboul, A. G.; Clifford, S.; Cioslowski, J.; Stefanov, B. B.; Liu, G.; Liashenko, A.; Piskorz, P.; Komaromi, I.; Martin, R. L.; Fox, D. J.; Keith, T.; Al-Laham, M. A.; Peng, C. Y.; Nanayakkara, A.; Challacombe, M.; Gill, P. M. W.; Johnson, B.; Chen, W.; Wong, M. W.; Gonzalez, C.; Pople, J. A. *Gaussian 03, Revision B.04*; Gaussian, Inc., Pittsburgh, PA.

[3] Frisch, M. J.; Trucks, G. W.; Schlegel, H. B.; Scuseria, G. E.; Robb, M. A.; Cheeseman, J. R.; Scalmani, G.; Barone, V.; Petersson, G. A.; Nakatsuji, H.; Li, X.; Caricato, M.; Marenich, A. V.; Bloino, J.; Janesko, B. G.; Gomperts, R.; Mennucci, B.; Hratchian, H. P.; Ortiz, J. V.; Izmaylov, A. F.; Sonnenberg, J. L.; Williams-Young, D.; Ding, F.; Lipparini, F.; Egidi, F.; Goings, J.; Peng, B.; Petrone, A.; Henderson, T.; Ranasinghe, D.; Zakrzewski, V. G.; Gao, J.; Rega, N.; Zheng, G.; Liang, W.; Hada, M.; Ehara, M.; Toyota, K.; Fukuda, R.; Hasegawa, J.; Ishida, M.; Nakajima, T.; Honda, Y.; Kitao, O.; Nakai, H.; Vreven, T.; Throssell, K.; Montgomery, J. A.; Peralta, Jr., J. E.; Ogliaro, F.; Bearpark, M. J.; Heyd, J. J.; Brothers, E. N.; Kudin, K. N.; Staroverov, V. N.; Keith, T. A.; Kobayashi, R.; Normand, J.; Raghavachari, K.; Rendell, A. P.; Burant, J. C.; Iyengar, S. S.; Tomasi, J.; Cossi, M.; Millam, J. M.; Klene, M.; Adamo, C.; Cammi, R.; Ochterski, J. W.; Martin, R. L.; Morokuma, K.; Farkas, O.; Foresman, J. B.; Fox, D. J. *Gaussian 16, Revision A.03*; Gaussian, Inc., Wallingford CT.

[4] Adamo, C.; Barone, V. Toward reliable density functional methods without adjustable parameters: The PBE0 model. *J. Chem. Phys.* **1999**, *110*, 6158−6170.

[5] Hehre, W. J.; Ditchfield, R.; Pople, J. A. Self-Consistent Molecular Orbital Methods. XII. Further Extensions of Gaussian-Type Basis Sets for Use in Molecular Orbital Studies of Organic Molecules. *J. Chem. Phys.* **1972**, *56*, 2257−2261.

[6] Clark, T.; Chandrasekhar, J.; Spitznagel, G. W.; Schleyer, P. V. R. Efficient diffuse function-augmented basis sets for anion calculations. III. The 3-21+G basis set for first-row elements, Li-F. *J. Comput. Chem.* **1983**, *4*, 294–301.

[7] Francl, M. M.; Pietro, W. J.; Hehre, W. J.; Binkley, J. S.; Gordon, M. S.; DeFrees, D. J.; Pople, J. A. Self-consistent molecular orbital methods. XXIII. A polarization-type basis set for second-row elements. *J. Chem. Phys.* **1982**, *77*, 3654−3665.

[8] Frisch, M. J.; Pople, J. A.; Binkley, J. S. Self-consistent molecular orbital methods 25. Supplementary functions for Gaussian basis sets. *J. Chem. Phys.* **1984**, *80*, 3265–3269.

[9] Krishnan, R.; Binkley, J. S.; Seeger, R.; Pople, J. A. Self-consistent molecular orbital methods. XX. A basis set for correlated wave functions. *J. Chem. Phys.* **1980**, *72*, 650–654.

[10] McLean, A. D.; Chandler, G. S. Contracted Gaussian basis sets for molecular calculations. I. Second row atoms, Z = 11−18. *J. Chem. Phys.* **1980**, *72*, 5639−5648.

[11] Spitznagel, G. W.; Clark, T.; von Ragué Schleyer, P.; Hehre, W. J. An evaluation of the performance of diffuse function-augmented basis sets for second row elements, Na-Cl. *J. Comput. Chem.* **1987**, *8*, 1109–1116.

[12] Ditchfield, R.; Hehre, W. J.; Pople, J. A. Self-Consistent Molecular-Orbital Methods. IX. An Extended Gausian-Type Basis for Molecular-Orbital Studies of Organic Molecules. *J. Chem. Phys.* **1971**, *54*, 724−728.

[13] Schaefer, A.; Horn, H.; Ahlrichs, R. Fully optimized contracted Gaussian-basis sets for atoms Li to Kr. *J. Chem. Phys.* **1992**, *97*, 2571−2577.

[14] Schaefer, A.; Huber, C.; Ahlrichs, R. Fully optimized contracted Gaussian-basis sets of triple zeta valence quality for atoms Li to Kr. *J. Chem. Phys*. **1994**, *100*, 5829−5835.

[15] Weigend, F.; Ahlrichs, R. Balanced basis sets of split valence, triple zeta valence and quadruple zeta valence quality for H to Rn: Design and assessment of accuracy. *Phys. Chem. Chem. Phys.* **2005**, *7*, 3297−3305.

[16] Dunning, T. H., Jr. Gaussian basis sets for use in correlated molecular calculations. I. The atoms boron through neon and hydrogen. *J. Chem. Phys.* **1989**, *90*, 1007−1023.

[17] Woon, D. E.; Dunning, T. H. Gaussian basis sets for use in correlated molecular calculations. III. The atoms aluminum through argon. *J. Chem. Phys.* **1993**, *98*, 1358–1371.

[18] Jensen, F. Polarization consistent basis sets: Principles. *J. Chem. Phys.* **2001**, *115*, 9113–9125.

[19] Jensen, F. Polarization consistent basis sets. II. Estimating the Kohn-Sham basis set limit. *J. Chem. Phys.* **2002**, *116*, 7372–7379.

[20] Jensen, F.; Helgaker, T. Polarization consistent basis sets. V. The elements Si-Cl. *J. Chem. Phys.* **2004**, *121*, 3463–3470.

[21] Andrae, D.; Haeussermann, U.; Dolg, M.; Stoll, H.; Preuss, H. Energy-adjusted *ab initio* pseudopotentials for the second and third row transition elements. *Theor. Chem. Acc.* **1990**, *77*, 123–141.

[22] Noro, T.; Sekiya, M.; Koga, T.; Saito, S. L. Relativistic contracted Gaussian-type basis functions for atoms K through Xe. *Chem. Phys. Lett.* **2009**, *481*, 229–233.

[23] Osanai, Y. O. U.; Sekiya, M.; Noro, T.; Koga, T. Valence and correlating basis sets for the second transition-metal atoms from Y to Cd. *Mol. Phys*. **2003**, *101*, 65–71.

[24] Andrae, D.; Häußermann, U.; Dolg, M.; Stoll, H.; Preuß, H. Energy-adjusted *ab initio* pseudopotentials for the second and third row transition elements. *Theor. Chim. Acta*. **1990**, *77*, 123–141.

[25] Weigend, F.; Ahlrichs, R. Balanced basis sets of split valence, triple zeta valence and quadruple zeta valence quality for H to Rn: Design and assessment of accuracy. *Phys. Chem. Chem. Phys.* **2005**, *7*, 3297−3305.

[26] Miertus, S.; Scrocco, E.; Tomasi, J. Electrostatic interaction of a solute with a continuum. A direct utilization of AB initio molecular potentials for the prevision of solvent effects. *Chem. Phys.* **1981**, *55*, 117−129.

[27] Hansen, A.E.; Bouman, T.D. Localized orbital/local origin method for calculation and analysis of NMR shieldings. Applications to ^{13}C shielding tensors. *J. Chem. Phys.* **1985**, *82*, 5035−5047.

[28] Pritchard, B. P.; Altarawy, D.; Didier, B.; Gibson, T. D.; Windus. T. L. A New Basis Set Exchange: An Open, Up-to-date Resource for the Molecular Sciences Community. *J. Chem. Inf. Model.* **2019**, *59*, 4814−4820.

[29] Portnoy, M.; Milstein, D. Chelate effect on the structure and reactivity of electron-rich palladium complexes and its relevance to catalysis. *Organometallics.* **1993**, *12*, 1655−1664.

[30] Raebiger, J. W.; Miedaner, A.; Curtis, C. J.; Miller, S. M.; Anderson, O. P.; DuBois, D. L. Using ligand bite angles to control the hydricity of palladium diphosphine complexes. *J. Am. Chem. Soc.* **2004**, *126*, 5502−5514.

[31] Nixon, J. F.; Sillett, G. J. ^{31}P and ^{195}Pt NMR studies on fluxional η^1-ligated 1,2,4-triphosphacyclopentadienyl palladium(II) and platinum(II) complexes. *J. Organomet. Chem.* **1993**, *461*, 237−245.

[32] Kane, J. C.; Wong, E. H.; Yap, G. P.; Rheingold, A. L. Synthesis and structural studies of molybdenum and palladium complexes of 1,5-diaza-3,7-diphosphacyclooctane ligands. *Polyhedron.* **1999**, *18*, 1183−1188.

[33] Gouygou, M.; Tissot, O.; Daran, J. C.; Balavoine, G. G. Biphosphole: A C_2 Symmetry Chiral Bidentate Ligand. Synthesis and Characterization Of Nickel, Palladium, Platinum, and Rhodium Complexes. *Organometallics.* **1997**, *16*, 1008−1015.

[34] Wächtler, E.; Priver, S. H.; Wagler, J.; Heine, T.; Zhechkov, L.; Bennett, M. A.; Bhargava, S. K. Metallophilic contacts in 2-$C_6F_4PPh_2$ bridged heterobinuclear complexes: a crystallographic and computational study. *Inorg. Chem.* **2015**, *54*, 6947−6957.

[35] David, M. A.; Wicht, D. K.; Glueck, D. S.; Yap, G. P.; Liable-Sands, L. M.; Rheingold, A. L. Palladium-catalyzed phosphaketene decarbonylation: diphosphaureylene intermediates in diphosphene formation. *Organometallics.* **1997**, *16*, 4768−4770.

[36] Sevillano, P.; Habtemariam, A.; Parsons, S.; Castiñeiras, A.; García, M. E.; Sadler, P. J. Gold (I)-induced chelate ring-opening of palladium (II) and platinum (II) triphos complexes. *J. Chem. Soc., Dalton Trans.* **1999**, *16*, 2861−2870.

[37] Zhuravel, M. A.; Grewal, N. S.; Glueck, D. S.; Lam, K. C.; Rheingold, A. L. Cyclometalation of Dimesitylphosphine in Cationic Palladium (II) and Platinum (II) Complexes: P− H *vs* C− H Activation. *Organometallics.* **2000**, *19*, 2882−2890.

[38] Mizuta, T.; Okano, A.; Sasaki, T.; Nakazawa, H.; Miyoshi, K. Palladium (II) and platinum (II) complexes of a tetraphosphamacrocycle. X-ray crystal structures of phosphorus analogs of a (tetramethylcyclam) metal complex. *Inorg. Chem.* **1997**, *36*, 200−203.

[39] Mason, L. J.; Perrault, E. M.; Miller, S. M.; Helm, M. L. Group 10 metal complexes of a cyclic diphosphine: The crystal structures of bis (cis-P,P′-diphenyl-1,4-diphospha-cyclohexane)M(II)chloride, M= palladium, platinum. *Inorg. Chem. Com.* **2006**, *9*, 946−948.

[40] Melaimi, M.; Thoumazet, C.; Ricard, L.; Le Floch, P. Syntheses of a 2,6-bis-(methylphospholyl) pyridine ligand and its cationic Pd(II) and Ni(II) complexes – application in the palladium-catalyzed synthesis of arylboronic esters. *J. Organomet. Chem.* **2004**, *689*, 2988−2994.

[41] Kohler, M. C.; Grimes, T. V.; Wang, X.; Cundari, T. R.; Stockland Jr, R. A. Arylpalladium phosphonate complexes as reactive intermediates in phosphorus − carbon bond forming reactions. *Organometallics.* **2009**, *28*, 1193−1201.

[42] Stockland, R. A.; Levine, A. M.; Giovine, M. T.; Guzei, I. A.; Cannistra, J. C. Reductive elimination from metal phosphonate complexes: Circumvention of competing protonolysis reactions. *Organometallics.* **2004**, *23*, 647−656.

[43] Stockland Jr, R. A.; Maher, D. L.; Anderson, G. K.; Rath, N. P. Demethylation of trimethylphosphite promoted by dichlorodiphosphineplatinum and palladium complexes. Structures of the metallophosphonate complexes $[Pt(P{\wedge}P)\{P(O)(OMe)_2\}_2]$($P{\wedge}P$= dppe, dppp). *Polyhedron*. **1999**, *18*, 1067−1075.

[44] Gómez-Ruiz, S.; Hey-Hawkins, E. Oxidative cleavage of tetraaryltetraphosphane-1, 4-diides by nickel (ii) and palladium (ii): formation of unusual Ni^0 and Pd^0 diaryldiphosphene complexes. *Dalton Trans.* **2007**, *48*, 5678−5683.

[45] Fenske, D.; Schottmueller, H. New Research of Reaction Behaviour of Triorganylcyclotriphosphines. The Crystal Structures of $[(PPh_3)_2Pt(P^tBu)_3]$, $[(PPh_3)_2Pd(P^tBu)_2$, $[(CO)_4Cr\{(P^iPr)_3\}_2]$, $[RhCl(PPh_3)(P^tBu)_3]$, $[(NiCO)_6(\mu_2\text{-}CO)_3\{(PtBu)_2\}_2]$, and $[(CpFeCO)_2(\mu\text{-}CO)(\mu\text{-}PH^tBu)]^+{\cdot}[FeCl_3(thf)]^-$. *Z. anorg. allg. Chem.* **1998**, *624*, 443−451.

[46] Phillips, I. G.; Ball, R. G.; Cavell, R. G. Reactions of perfluoromethyl-substituted cyclopolyphosphines with zerovalent group 10 metal complexes. Crystal and molecular structure of a complex with a coordinated diphosphene, $[Pd(\eta^2\text{-}CF_3P{=}PCF_3)(PPh_3)_2]$. *Inorg. Chem.* **1992**, *31*, 1633−1641.

[47] Chatt, J.; Hitchcock, P. B.; Pidcock, A.; Warrens, C. P.; Dixon, K. R. The nature of the co-ordinate link. Part 11. Synthesis and phosphorus-31 nuclear magnetic resonance spectroscopy of platinum and palladium complexes containing side-bonded (*E*)-diphenyldiphosphene. X-Ray crystal and molecular structures of $[Pd\{(E)\text{-}PhP{=}PPh\}(Ph_2PCH_2CH_2PPh_2)]$ and $[Pd\{[(E)\text{-}PhP{=}PPh][W(CO)_5]_2\}(Ph_2PCH_2CH_2PPh_2)]$. *J. Chem. Soc., Dalton Trans.* **1984**, *10*, 2237−2244.
